# *TERT* promoter status and gene copy number gains: effect on *TERT* expression and association with prognosis in breast cancer

**DOI:** 10.18632/oncotarget.20560

**Published:** 2017-08-24

**Authors:** Mathilde Gay-Bellile, Lauren Véronèse, Patricia Combes, Eleonore Eymard-Pierre, Fabrice Kwiatkowski, Marie-Mélanie Dauplat, Anne Cayre, Maud Privat, Catherine Abrial, Yves-Jean Bignon, Marie-Ange Mouret-Reynier, Philippe Vago, Frédérique Penault-Llorca, Andrei Tchirkov

**Affiliations:** ^1^ Université Clermont Auvergne, INSERM, U1240 Imagerie Moléculaire et Stratégies Théranostiques, F-63000 Clermont Ferrand, France; ^2^ Service de Cytogénétique Médicale, CHU Clermont-Ferrand, F-63003 Clermont-Ferrand, France; ^3^ Département de Recherche Clinique, Centre Jean Perrin, F-63011 Clermont-Ferrand, France; ^4^ Département de Pathologie, Centre Jean Perrin, F-63011 Clermont-Ferrand, France; ^5^ Département d’Oncogénétique, Centre Jean Perrin, F-63011 Clermont-Ferrand, France; ^6^ Centre de Ressources Biologiques BB-0033-00075, Centre Jean Perrin, F-63011 Clermont-Ferrand, France

**Keywords:** breast cancer, *TERT* promoter mutation and polymorphism, *TERT* gene copy number gains, *MYC* overexpression, prognosis

## Abstract

Upregulation of the telomerase reverse transcriptase (*TERT*) gene in human cancers leads to telomerase activation, which contributes to the growth advantage and survival of tumor cells. Molecular mechanisms of *TERT* upregulation are complex, tumor-specific and can be clinically relevant. To investigate these mechanisms in breast cancer, we sequenced the *TERT* promoter, evaluated *TERT* copy number changes and assessed the expression of the *MYC* oncogene, a known transcriptional *TERT* regulator, in two breast cancer cohorts comprising a total of 122 patients. No activating *TERT* promoter mutations were found, suggesting that this mutational mechanism is not likely to be involved in *TERT* upregulation in breast cancer. The T349C promoter polymorphism found in up to 50% of cases was not correlated with *TERT* expression, but T349C carriers had significantly shorter disease-free survival. *TERT* gains (15-25% of cases) were strongly correlated with increased *TERT* mRNA expression and worse patient prognosis in terms of disease-free and overall survival. Particularly aggressive breast cancers were characterized by an association of *TERT* gains with *MYC* overexpression. These results evidence a significant effect of gene copy number gain on the level of *TERT* expression and provide a new insight into the clinical significance of *TERT* and *MYC* upregulation in breast cancer.

## INTRODUCTION

The telomerase reverse transcriptase (*TERT*) gene encodes the catalytic subunit of telomerase that maintains telomere length. Telomerase activity occurs in more than 90% of cancers [1]. One of the hallmark features of tumor cells, it contributes to their growth advantage and survival, and upregulation of the *TERT* gene is the major mechanism of telomerase activation in human cancer [2]. In breast tumors, *TERT* overexpression was associated with tumor aggressiveness [3] and poor survival after adjuvant [4] and neoadjuvant [5] chemotherapy.

Several molecular events can modify *TERT* expression in cancer cells. Somatic mutations and functional single nucleotide polymorphisms (SNP) in the *TERT* promoter can lead to changes in the expression of the gene [6]. Overexpression of the oncogene *MYC* can directly enhance *TERT* expression by increased binding to the *TERT* promoter [7]. Increased copy number or amplification of the *TERT* gene can also result in *TERT* upregulation [8–10].

The *TERT* promoter is the most important element of telomerase expression because it harbors binding sites for numerous cellular transcriptional activators and repressors that directly or indirectly regulate gene expression. Recurrent somatic mutations in the *TERT* promoter were found in cancers of the central nervous system (43%), bladder (59%), thyroid (follicular cell-derived, 10%) and skin (melanoma, 29%) [11, 12]. In particular, two hotspot C228T and C250T mutations create binding motifs for transcription factor ETS2 and increase *TERT* transcriptional activity [13]. In addition, the T349C SNP, chr5:1,295,349 T>C (rs2853669) affects telomerase activity and telomere length [14, 15]. This polymorphism can be studied in both blood and tumor samples since the results are concordant [14, 16].

In breast cancer, only limited data are available concerning the incidence of mutations and SNP in the *TERT* promoter, *TERT* gains and their relationship with *TERT* upregulation and patient prognosis. To address these issues, we investigated the mutational status and genotype of the *TERT* promoter, *TERT* gene copy number and *TERT* and *MYC* mRNA expression in two breast cancer cohorts. The first cohort comprised 77 patients with triple-negative breast cancer (TNBC) from two recent neoadjuvant trials assessing the efficacy and toxicity of anti-EGFR antibodies combined with chemotherapy [17, 18]. The patients had been followed for 5 years. The second cohort consisted of a retrospective series of 45 patients treated with standard neoadjuvant chemotherapy (NCT). Most of them had common hormone-positive tumors and had been followed for more than 10 years.

## RESULTS

### *TERT* promoter status

We detected no activating *TERT* promoter mutations, including hotspot mutations C228T and C250T, in 77 pre-NCT breast tumor biopsies (cohort #1) and 45 post-NCT residual tumors (cohort #2). The T349C (rs2853669) SNP was identified in 42.3% of tumor biopsies and 48.9% of residual tumors (Table 1). In both #1 and #2 cohorts, most tumors with the variant C allele had a heterozygous 349 T/C genotype (37.2% and 44.4%, respectively) whereas the frequency of 349 C/C homozygotes was low (5.1% and 6.7%). Overall, the frequencies of T349C in the two cohorts were similar to those in the 1000 Genome database for Europe.

**Table 1:** Distributions of *TERT* promoter rs2853669 alleles (T349C SNP) in breast cancer cohorts (with 95%-confidence intervals) and 1000 genome database

	349 T/T	349 T/C	349 C/C
Breast cancer cohort #1			
pre-NCT biopsies (n= 77)	57.7% (46.7-68.7%)	37.2% (26.5-47.9%)	5.1% (1.1-12.3%)
**Breast cancer cohort #2**			
post-NCT residual tumors (n= 45)	48.9% (33.4-63.5%)	44.4% (29.9-58.9%)	6.7% (1.7-18.4%)
**1000 Genome Database**			
All populations	53%	34.5%	12.5%
European population	52.1%	38.2%	9.7%

### *TERT* copy number aberrations

We found that *TERT* gains were relatively frequent events in both cohorts. Twenty tumors (25.6%) in cohort #1 and seven tumors (15.6%) in cohort #2 had *TERT* gains. *TERT* losses were rare, with the alteration occurring in three tumors (3.9%) in cohort #1 and two tumors (4.4%) in cohort #2.

### Associations of the T349C genotype and *TERT* gene copy number gains with *TERT* and *MYC* expression

**Figure 1:**
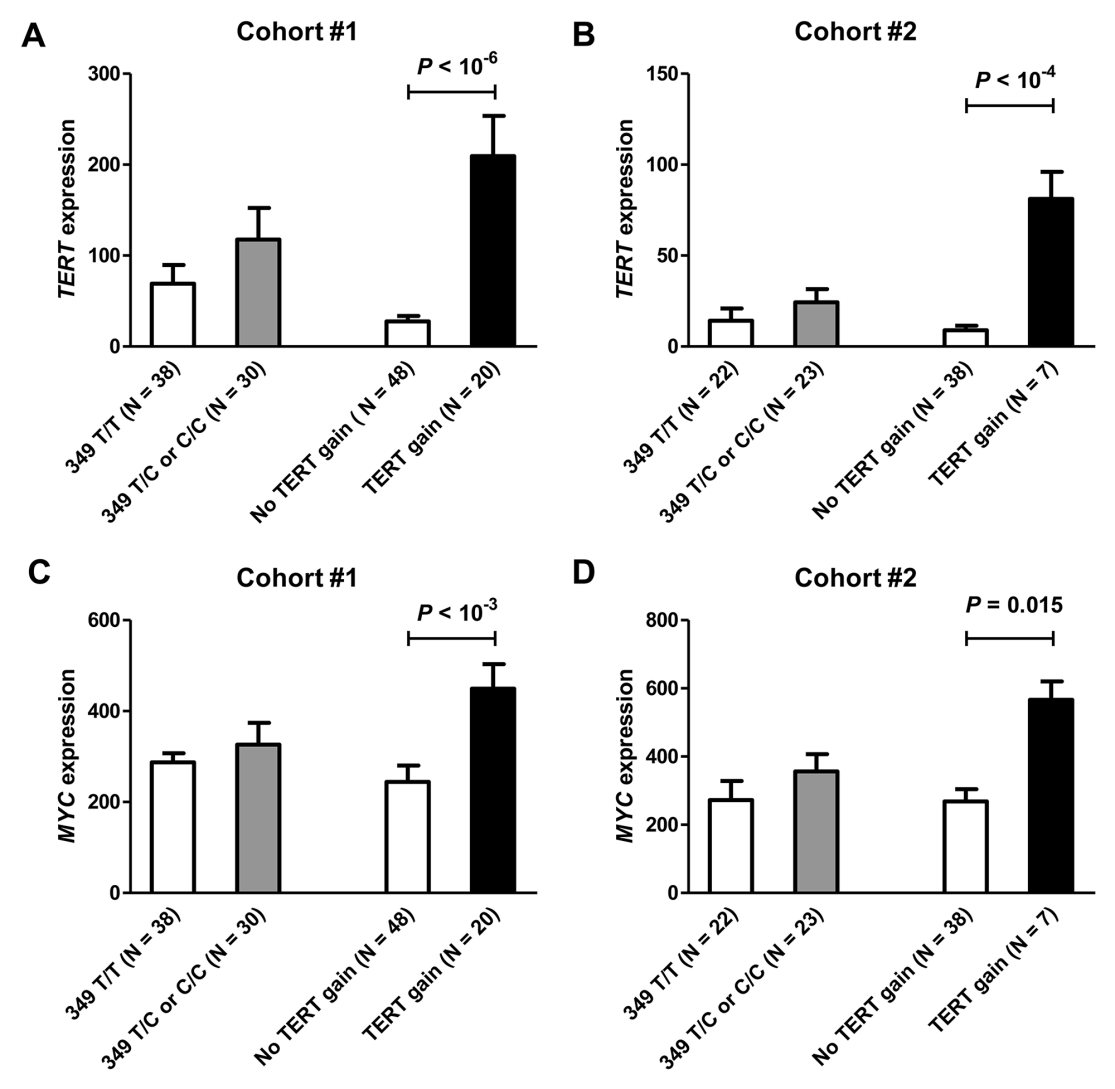
The levels of *TERT* and *MYC* gene expression according to T349C status and *TERT* gene copy number in breast cancer cohorts #1 **(A, C)** and #2 **(B, D)**. *TERT* gains were associated with a significant increase in *TERT* and *MYC* expression in both cohorts.

We investigated whether T349C genotype and *TERT* gains were associated with changes in the expression of the *TERT* gene (Figure 1A–1B). The results were similar in the two cohorts. Tumors with 349 T/C or C/C polymorphism had increased expression of *TERT*, but the difference from the 349 T/T cases was not significant. In contrast, *TERT* gene copy number gains were associated with a highly significant increase in gene expression as compared to cases without *TERT* gain. Of note, *TERT* gains were more frequent in the presence of the T349C SNP. In cases with 349 T/C or C/C genotypes, the relative risk of a *TERT* gain increased 3.2-fold (*P* = 0.0036, 95% confidence interval from 1.5 to 6.9) in cohort #1 and 5.7-fold in cohort #2 (*P* = 0.11, 95% confidence interval 1.0-32.0), as compared to the risk in 349 T/T cases.

In parallel, we investigated if the presence of *TERT* T349C polymorphism or gain was associated with the expression levels of *MYC* oncogene, a strong positive regulator of *TERT* expression (Figure 1C–1D). We found no association between the presence of the T349C SNP and *MYC* expression. In contrast, a significantly higher expression of the *MYC* gene were observed in cases with *TERT* gains (cohort #1, *P* < 10^-3^; cohort #2, *P* = 0.015). Overall, the levels of *TERT* and *MYC* expression were significantly correlated in both cohorts (Pearson *r* = 0.696, *P* < 10^-7^ in cohort #1 and *r* = 0.542, *P* < 10^-3^ in cohort #2).

### Clinical significance of the T349C SNP and *TERT* gene copy number gains

**Figure 2:**
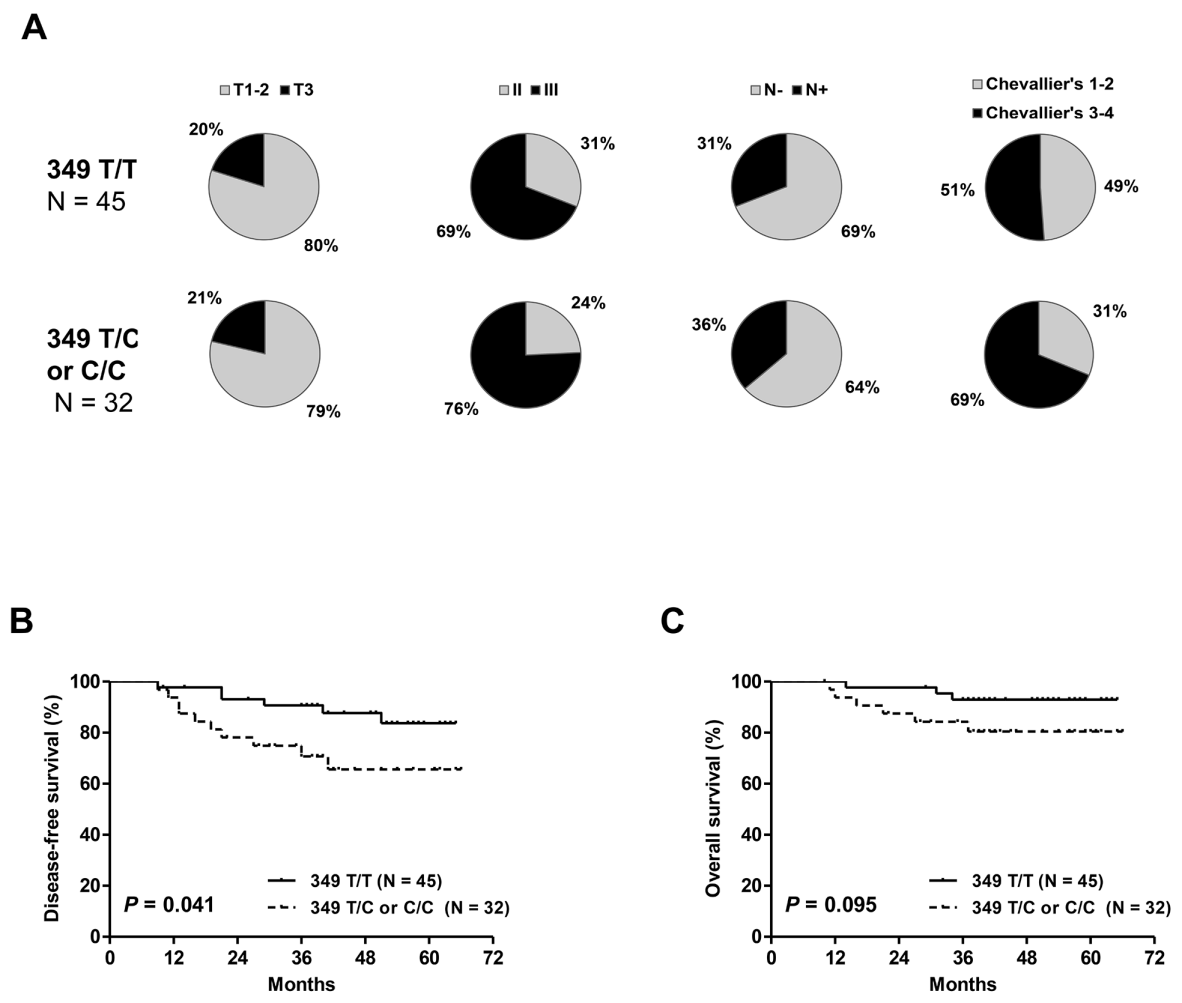
Distribution of clinicopathologic characteristics: tumor size, tumor grade, nodal status and pathologic response **(A)** disease-free **(B)** and overall survival **(C)** in cohort #1 according to T349C status. Patients with 349 T/C or C/C alleles had significantly shorter DFS and a trend toward a shorter OS.

The presence of the T349C SNP (349 T/C and C/C genotypes) in cohort #1 was not significantly associated with clinicopathologic characteristics such as tumor size, tumor grade, nodal status and pathologic response to NCT (Figure 2A). However, the rate of incomplete pathologic responses tended to be higher in T349C cases than in patients with T349T (69% vs. 51%, *P* = 0.12). Patients with the T349C genotype had significantly shorter DFS and a trend toward a shorter OS (Figure 2B–2C).

**Figure 3:**
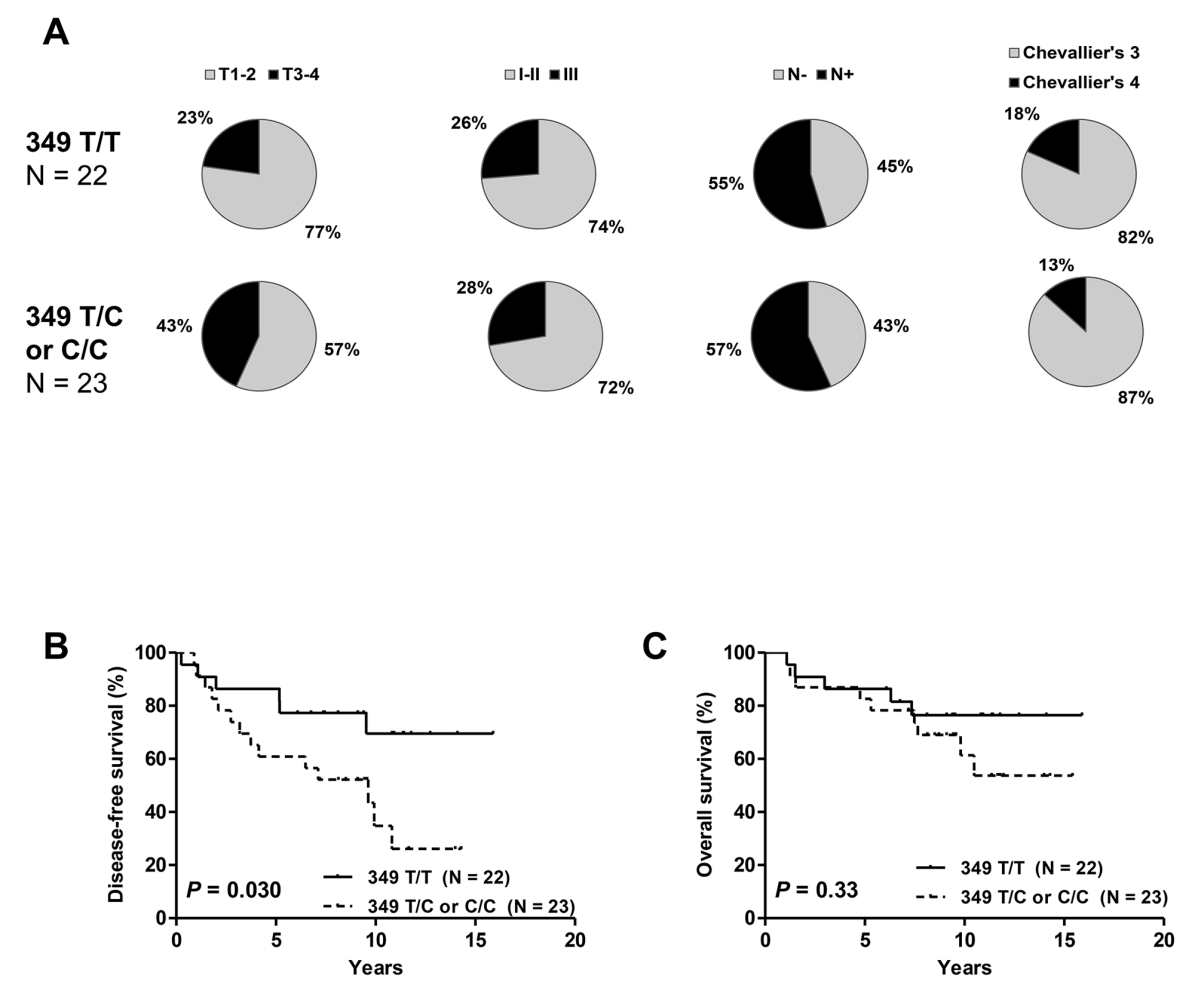
Distribution of clinicopathologic characteristics: tumor size, tumor grade, nodal status and pathologic response **(A)** disease-free **(B)** and overall survival **(C)** in cohort #2 according to T349C status. Patients with 349 T/C or C/C alleles had significantly shorter DFS but no difference in OS.

In cohort #2, the T349C SNP was not associated with clinicopathologic characteristics (Figure 3A). T3-4 tumors were more frequent in T349C patients than in those with 349 T/T genotype (43% vs 23%), but not to a level of significance (*P* = 0.14). Survival analysis showed, as in cohort #1, a significant effect of T349C on DFS but not on OS (Figure 3B).

**Figure 4:**
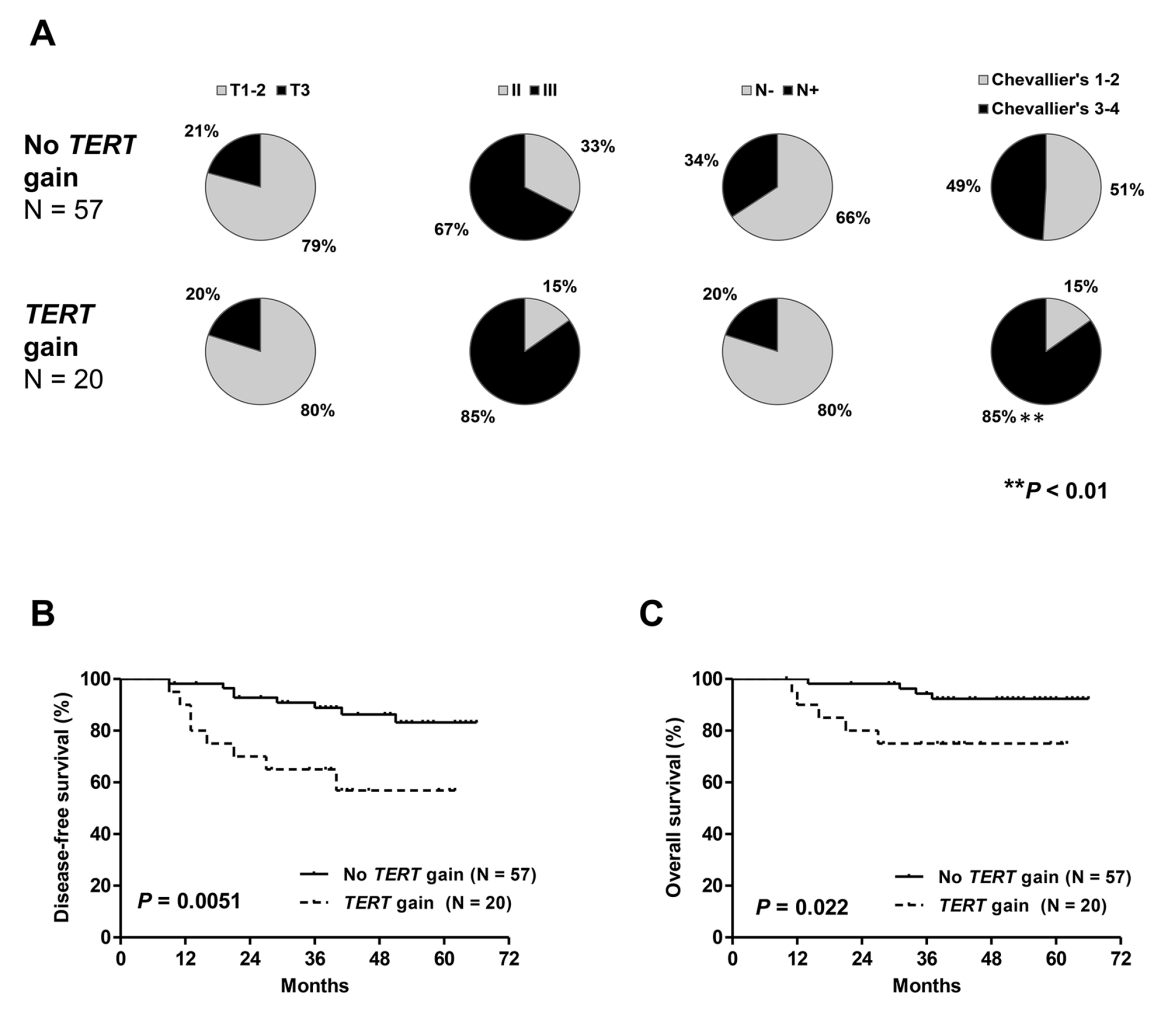
Distribution of clinicopathologic characteristics: tumor size, tumor grade, nodal status and pathologic response **(A)** disease-free **(B)** and overall survival **(C)** in patients from cohort #1 with and without *TERT* gene copy number gain. Patients with *TERT* gains had a significantly higher rate of incomplete pathologic responses and significantly shorter DFS and OS.

Patients with *TERT* gains in cohort #1 had a trend toward a higher proportion of SBR grade III tumors (*P* = 0.13) and a significantly higher rate of incomplete pathologic responses (*P* < 0.01, Figure 4A). No association with other clinicopathologic characteristics was observed. Both DFS and OS were significantly shorter in patients with *TERT* gains (Figure 4B–4C).

**Figure 5:**
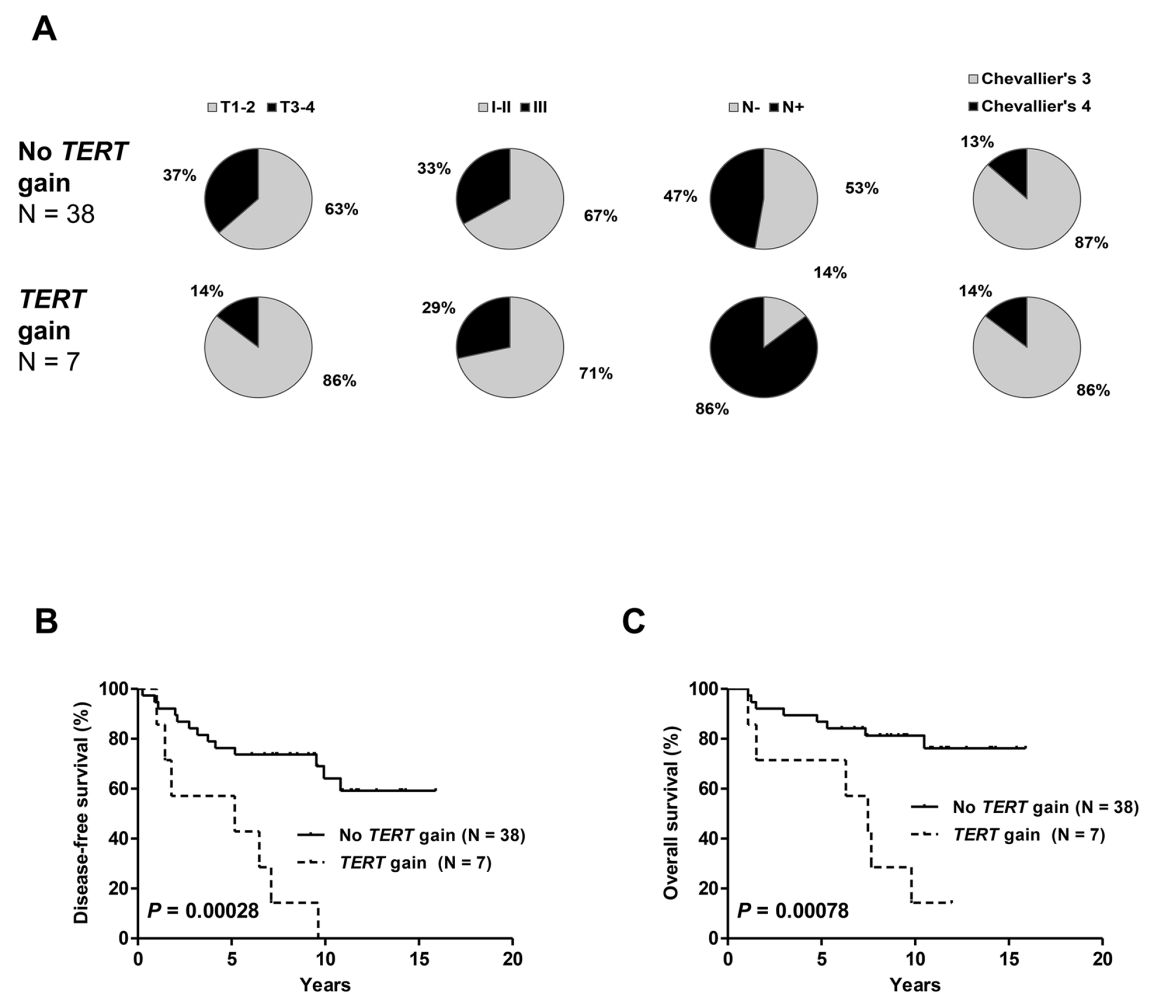
Distribution of clinicopathologic characteristics: tumor size, tumor grade, nodal status and pathologic response **(A)** disease-free **(B)** and overall survival **(C)** in patients from cohort #2 with and without *TERT* gene copy number gain. Patients with *TERT* gains had a significantly higher rate of incomplete pathologic responses and significantly shorter DFS and OS.

In cohort #2, the number of cases with *TERT* gains was small (n=7) and the distribution of clinicopathological features among cases with and without gains was not significantly different (Figure 5A). However, the occurrence of a *TERT* gain was significantly related to shorter DFS and OS (Figure 5B–5C).

### *MYC* expression levels impact the prognostic value of *TERT* gains

**Figure 6:**
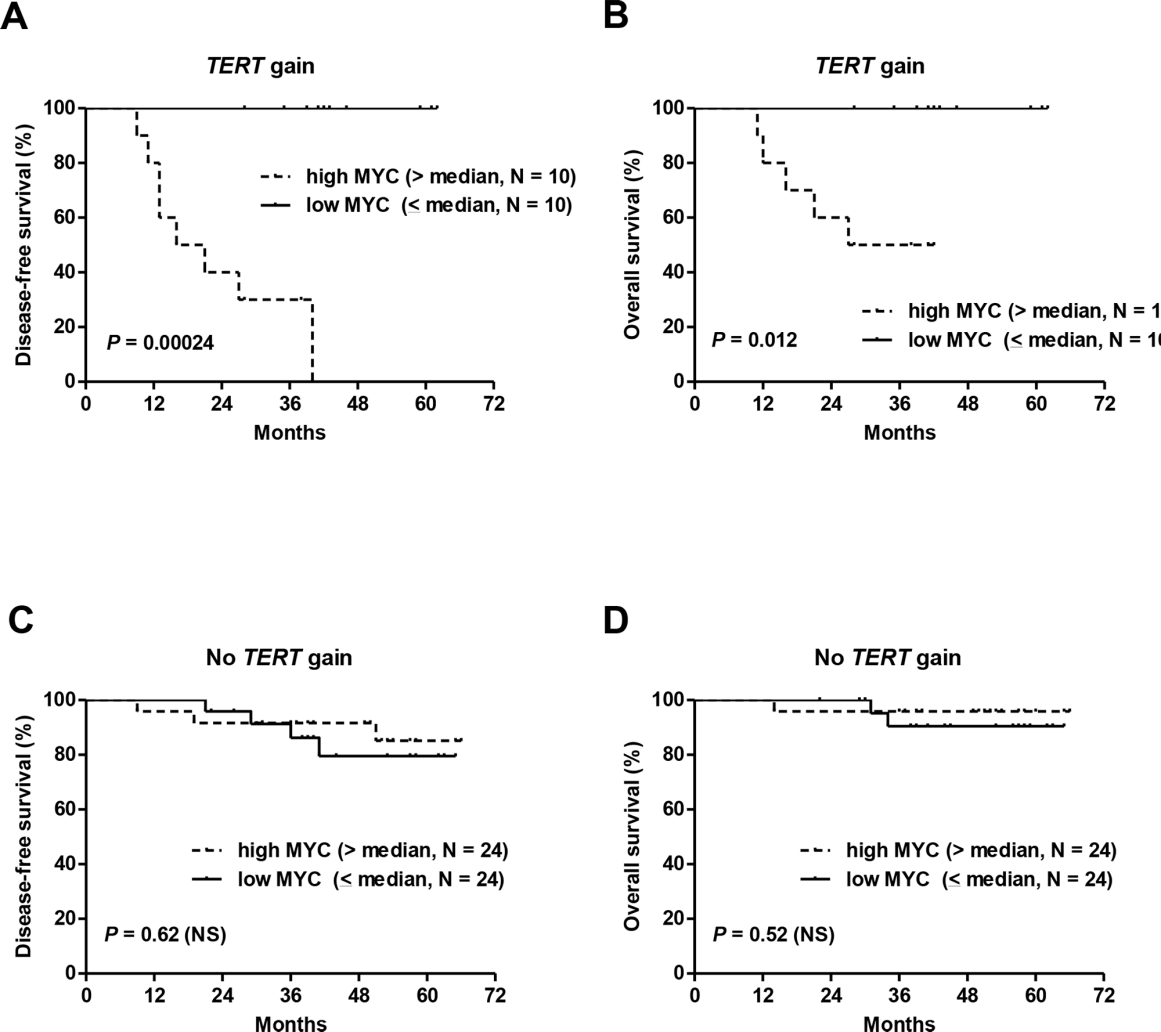
The combination of *TERT* gene copy number gains and *MYC* overexpression was associated with the shortest DFS and OS in cohort #1 Lower *MYC* levels annulled the negative effect of *TERT* gains on DFS and OS **(A, B)**. In cases without *TERT* gains, *MYC* levels had no effect on patient survival **(C, D)**.

Univariate Cox analysis for DFS in cohort #1 showed that the presence of the T349C genotype and *TERT* gain increased significantly the risk of tumor recurrence with HR of 2.8 and 3.8, respectively (Table 2). High *MYC* expression (> median) also had a significant and negative effect on DFS (HR of 3.5). Importantly, very high levels of *MYC* expression (upper quartile, > 421) were associated with a much higher risk of tumor recurrence (HR of 9.4, 95%-CI: 3.2-27.2, *P* < 0.0001). As expected, incomplete pathologic responses to NCT according to Chevallier's classification had a significant negative prognostic effect (HR of 3.6). When adjusted for pathologic responses, *TERT* gain and *MYC* overexpression remained significant prognostic factors (Table 2). As mentioned above, tumors with *TERT* gain frequently had *MYC* overexpression, and it was assumed that the presence of both abnormalities would confer a worse prognosis. Indeed, the combination of *MYC* overexpression with *TERT* gains was associated with the shortest DFS and OS, whereas lower *MYC* levels annulled any negative effect of *TERT* gain on DFS and OS (Figure 6A–6B). In cases without *TERT* gain, *MYC* levels were not prognostic (Figure 6C–6D).

**Table 2:** Cox analysis for disease-free survival in breast cancer cohort #1

Parameters	Unadjusted		Adjusted*	
	HR (95% CI)	*P*	HR (95% CI)	*P*
*TERT* gain (yes vs no)	3.8 (1.4-10.3)	0.0078	3.0 (1.1-8.4)	0.032
*TERT* T349C (yes vs no)	2.8 (1.0-7.7)	0.047	2.3 (0.9-6.6)	0.096
*MYC* (high vs low)**	3.5 (1.1-10.8)	0.031	3.2 (1.1-10.1)	0.045
Chevallier's classification (3-4 vs 1-2)	3.6 (1.1-12.6)	0.048	-	-

*Adjusted for treatment response according to Chevallier's classification: incomplete pathologic response, classes 3-4, vs complete pathologic response, classes 1-2.

**High > median, low < median (181)

Hazard ratio, HR; 95% confidence interval, 95% CI.

## DISCUSSION

Telomerase activation protects malignant cells from apoptosis and senescence by preventing further telomere loss [2, 3, 19]. Regulation of telomerase activation in cancer is complex and multifactorial and involves transcriptional activation of the *TERT* gene [10].

Activating somatic mutations of the *TERT* promoter have been reported in various cancers [11, 12]. The two most common mutations, C228T and C250T, map -124 and -146 bp upstream of the *TERT* ATG site (chr5: 1,295,228 C>T and 1,295,250 C>T, respectively) and create binding sites for ETS/TCF transcription factors, which leads to a two- to four-fold increase in transcriptional activity [13, 20]. These mutations were not found in limited breast cancer series [11, 12]. Our results from a total of 122 breast tumors evidenced no *TERT* promoter mutations, suggesting that this mutational mechanism is not likely to be involved in *TERT* upregulation in breast cancer.

The variant C-allele of the rs2853669 (T349C) polymorphism disrupts an ETS2-binding site (EtsA) in the proximal region of the *TERT* promoter [14]. Although decreased binding of transcription factor ETS2 can modify the expression of the gene, opinions differ on its effect on the level of *TERT* expression in cancer cells. Our results from cases of breast cancer did not show any significant effect of the T349C SNP on *TERT* expression. Previous studies showed an association of the T349C genotype with reduced *TERT* expression in non-small cell lung cancer, bladder cancer and glioblastoma [16, 21, 22]. However, a recent report on four hepatocellular carcinoma cell lines described increased luciferase *TERT* promoter activity in the presence of the T349C SNP [23]. The T349C variant site is close to (2 bp downstream) the binding site of transcriptional repressor E2F1, and T/C and C/C variants seem to inhibit E2F1 binding to the *TERT* promoter. In the presence of activating *TERT* mutations, this SNP was suggested to enhance *TERT* transcription levels by blocking E2F1 binding to its promoter. These recent findings suggest that the cooperation of native and changed (mutations, SNP) binding sites for multiple transcriptional factors in the *TERT* promoter could regulate *TERT* expression and result in cancer-specific effects of SNP.

There has been much debate about the clinical effect of the T349C SNP on the survival of patients with malignancies. Several studies reported its positive prognostic value in the context of activating *TERT* promoter mutations in bladder cancer and clear cell renal cell carcinoma [22, 24, 25]. However, more recent reports have shown that this SNP has a negative effect in glioblastoma and liver cancer patients bearing activating *TERT* promoter mutations [23, 26]. Among acute myeloid leukemia patients without *TERT* promoter mutations, T349C carriers had significantly shorter survival times [27]. Our study shows for the first time a negative effect of the T349C SNP on survival in breast cancer patients in the absence of activating somatic mutations. However, this SNP was significantly associated with *TERT* copy number gains in our patient series.

Increased *TERT* gene dosage was seen in about 30% of various human tumor-derived cell lines, including breast cancer cells [28]. We found *TERT* copy number gains in more than 20% of primary breast tumors examined. Previous studies yielded conflicting results regarding the effect of *TERT* gain on *TERT* mRNA expression. *TERT* gene gains were associated with higher *TERT* mRNA expression in non-small-cell lung adenocarcinoma and Merkell cell carcinoma [29, 30] and with high TERT protein expression in breast cancer and cervical carcinomas [28, 31]. However, no significant association was found in colorectal carcinomas and squamous-cell lung cancer [30, 32]. Our observation of a highly positive correlation between the *TERT* gene copy number and its mRNA level suggests that *TERT* gain has a functional effect on *TERT* transcription in breast cancer.

We showed that *TERT* gains identified in pre-NCT biopsies were predictive of incomplete pathological responses and shorter survival. In addition, *TERT* gain was a significant predictor of worse survival when assessed in residual post-NCT tumors. To our knowledge, only two studies have reported a negative effect of *TERT* gains on survival in non-small cell lung carcinomas and melanomas [30, 33]. For the first time, we show that *TERT* gain is also a negative prognostic marker associated with higher risk of disease recurrence or death from breast cancer and could potentially be used to adapt treatment strategies.

*TERT* upregulation correlates with increased telomerase activity. Inhibition of telomerase in breast tumors might potentiate the effects of standard anti-cancer treatments [34]. This opens up the perspective of using telomerase inhibitors in patients resistant to NCT. Preclinical studies with the telomerase inhibitor GRN163L showed that it can restore sensitivity of HER2+ breast cancer cell lines to trastuzumab and so patients with HER2+ breast cancers were recruited into a phase I clinical trial (NCT 01265927). In combination with chemotherapy, GRN163L was used in a phase II clinical trial in patients with locally recurrent or metastatic breast cancer (NCT01256762). These clinical trials have been completed but the study results are not yet available [34].

Telomerase reactivation and/or *TERT* upregulation correlate with *MYC* overexpression [35, 36]. In line with these findings, we observed that *MYC* and *TERT* expression levels are significantly correlated. MYC is a primary transcription factor of the *TERT* gene and stimulates its promoter activity [37, 38]. In breast cancer cells, MYC binds to an ETS2 site (EtsA) in the *TERT* gene promoter and increases *TERT* expression levels [15]. As mentioned above, this binding site is disrupted by T349C variants, but we observed no significant changes in *TERT* or *MYC* expression according to the presence or absence of this SNP. In contrast, *MYC* overexpression was strongly associated with the presence of *TERT* gains. Interestingly, a recent study showed that TERT regulates MYC ubiquitination, stabilization and binding to its target gene promoters [39]. This feed-forward transcriptional loop between MYC and TERT is functionally critical for oncogenesis. In our study, we found that *MYC* overexpression often coincided with *TERT* gains. Enhanced MYC stability and function in cells with high TERT and MYC levels could potentiate MYC-dependent oncogenesis.

By combining the data on *TERT* gain and *MYC* overexpression we were able to isolate a particularly bad prognosis subgroup of breast cancer patients. MYC is considered to promote tumor cell survival, proliferation and progression in breast cancer [40, 41] and regulates the expression of 13 different poor-outcome cancer signatures [42]. We found that *MYC* overexpression had a negative prognostic value in the whole patient population and a particularly significant effect within the subgroup of patients with *TERT* gains. A concomitant upregulation of *TERT* and *MYC* identified patients with a high risk of breast cancer recurrence. In contrast, lower *MYC* expression levels were associated with a favorable prognosis, despite the presence of *TERT* gains.

In conclusion, we found no activating *TERT* promoter mutations in 122 breast cancer patients. The T349C *TERT* promoter SNP was not significantly associated with *TERT* expression but T349C carriers had shorter survival. Notably, *TERT* gene copy number gain was significantly related to *TERT* upregulation and, in association with *MYC* overexpression, characterized particularly aggressive disease. These results show a significant effect of gene copy number gain on *TERT* expression level and provide a new insight into the clinical significance of *TERT* and *MYC* upregulation in breast cancer.

## MATERIALS AND METHODS

### Patients and samples

Cohort #1 comprised 77 patients with operable, stage II–III TNBC enrolled in two neoadjuvant trials evaluating the efficacy and toxicity of anti-EGFR antibodies plus chemotherapy: panitumumab combined with a standard neoadjuvant anthracycline/taxane-based [17] and cetuximab combined with docetaxel [18]. After NCT, pathologic complete responses (classes 1 and 2 according to Chevallier's classification) were observed in 32 of 77 cases (41.6%). The remaining 45 (58.4%) patients had incomplete pathologic responses (class 3, N = 39 and class 4, N = 6). The median follow-up in this cohort was 3.8 years (range, 0.9-5.5 years). During follow-up, 16 (20.8%) patients relapsed and 11 (14.3%) died from breast cancer. Cohort #2 consisted of archived samples of residual post-NCT tumors from 45 women diagnosed with invasive breast cancer and treated with NCT protocols [43] between 1996 and 2010 at the Jean Perrin Cancer Center (Clermont-Ferrand, France). Following NCT, patients underwent appropriate surgery and radiotherapy. Those with important residual disease received adjuvant chemotherapy. Menopausal patients with hormone receptor-positive tumors received tamoxifen for 5 years. The mean follow-up was 9.1 years (range, 1.1-15.9 years). During this period, 20 (44.4%) patients relapsed and 14 (31.1%) died from breast cancer. In cohort #1, the study was performed on pre-treatment tumor biopsies and in cohort #2 on post-NCT residual tumor samples cryopreserved in the Biological Resources Center BB-0033-00075. DNA and RNA were simultaneously extracted with the AllPrep DNA/RNA/miRNA Universal Kit (Qiagen, Courtaboeuf, France) according to the manufacturer's instructions. The study was approved by the institutional review board.

### Gene expression analysis

Total RNA was converted to cDNA by reverse transcription with Superscript II reverse transcriptase (Invitrogen, Cergy-pontoise, France) according to the manufacturer's instructions. The expression of *TERT* was quantified by real-time RT-PCR in the Lightcycler 480 System (Roche Diagnostics, Meylan, France) as described previously [44, 45]. *MYC* was quantified with primers described in the literature [46]. The normalized copy numbers were expressed as the ratio between transcript copy numbers of the target and control (*B2M*) genes multiplied by 100.

### *TERT* promotor sequencing

The amplification of genomic DNA for the *TERT* promoter region (267 pb) was performed with the following primers: forward 5’-CCGGGCTCCCAGTGGATT-3’ and reverse 5’-TTCCCACGTGCGCAGCAG-3’. PCR conditions consisted of an initial heating at 95°C for 15 minutes followed by 40 cycles at 95°C for 30 seconds, 60°C for 20 seconds and 72°C for 30 seconds. Amplified PCR products were sequenced with BigDye v1.1 on the ABI 3500 Genetic Analyzer (Applied Biosystems, Courtaboeuf, France).

### *TERT* copy number quantification

*TERT* gene copy number was quantified by Quantitative Multiplex Fluorescent Polymerase Chain Reaction (QMF-PCR) analysis. QMF-PCR was performed on a TProfessional thermocycler (Biometra, Archamps, France) as previously described [47, 48]. Primers were designed for three fragments of the *TERT* gene on chromosome 5p (5’UTR, Exon 9 and 3’UTR) to cover the entire gene. The 5’UTR region is very GC-rich and was difficult to amplify. We therefore had to limit the analysis to exon 9 and 3’UTR. Seven genes on chromosomes 2p, 4p, 7q, 10q 11p, 11q (*PVRL1, BOD1L, RET, ZNF638, AGBL2, CFTR* and *POR*) were co-amplified as controls. Primers and technical details are described in Supplementary Methods. The fluorescence intensities of PCR products were correlated with the copy number of the relevant exons. Fourteen control DNAs were included in each experiment: 12 normal DNAs and 3 DNAs with known *TERT* copy number (loss, normal and gain, as determined by array-comparative genomic hybridization). A dosage quotient was calculated relative to all the other amplified exons in patients and controls. The range of ratios corresponding to two copies of *TERT* gene was set between 0.8 and 1.2 (mean +/- 3 standard deviations of values obtained in normal DNA samples). Ratios > 1.2 were considered as gains and < 0.8 as losses.

### Statistical analysis

Standard tests (Kruskal-Wallis H test, ANOVA, Student's t-test, chi-squared test, Pearson's correlation) were used to study the relationship between characteristics. Disease-free survival (DFS) was measured from diagnosis until the first relapse (local or distant) and overall survival (OS) from the start of NCT until the last follow-up report. Survival curves were established by the Kaplan-Meier method and compared with the log-rank test.

## SUPPLEMENTARY MATERIALS FIGURES AND TABLES



## References

[1] Shay JW, Bacchetti S (1990). A survey of telomerase activity in human cancer. Eur J Cancer Oxf Engl.

[2] Hanahan D, Weinberg RA (2011). Hallmarks of cancer: the next generation. Cell.

[3] Poonepalli A, Banerjee B, Ramnarayanan K, Palanisamy N, Putti TC, Hande MP (2008). Telomere-mediated genomic instability and the clinico-pathological parameters in breast cancer. Genes Chromosomes Cancer.

[4] Lu L, Zhang C, Zhu G, Irwin M, Risch H, Menato G, Mitidieri M, Katsaros D, Yu H (2011). Telomerase expression and telomere length in breast cancer and their associations with adjuvant treatment and disease outcome. Breast Cancer Res BCR.

[5] Gay-Bellile M, Romero P, Cayre A, Véronèse L, Privat M, Singh S, Combes P, Kwiatkowski F, Abrial C, Bignon YJ, Vago P, Penault-Llorca F, Tchirkov A (2016). ERCC1 and telomere status in breast tumours treated with neoadjuvant chemotherapy and their association with patient prognosis. J Pathol Clin Res.

[6] Liu T, Yuan X, Xu D (2016). Cancer-Specific Telomerase Reverse Transcriptase (TERT) Promoter Mutations: Biological and Clinical Implications. Genes.

[7] Wu KJ, Grandori C, Amacker M, Simon-Vermot N, Polack A, Lingner J, Dalla-Favera R (1999). Direct activation of TERT transcription by c-MYC. Nat Genet.

[8] Cao Y, Bryan TM, Reddel RR (2008). Increased copy number of the TERT and TERC telomerase subunit genes in cancer cells. Cancer Sci.

[9] Hahn WC, Meyerson M (2001). Telomerase activation, cellular immortalization and cancer. Ann Med.

[10] Zhu J, Zhao Y, Wang S (2010). Chromatin and epigenetic regulation of the telomerase reverse transcriptase gene. Protein Cell.

[11] Killela PJ, Reitman ZJ, Jiao Y, Bettegowda C, Agrawal N, Diaz LA, Friedman AH, Friedman H, Gallia GL, Giovanella BC, Grollman AP, He TC, He Y (2013). TERT promoter mutations occur frequently in gliomas and a subset of tumors derived from cells with low rates of self-renewal. Proc Natl Acad Sci U S A.

[12] Vinagre J, Almeida A, Pópulo H, Batista R, Lyra J, Pinto V, Coelho R, Celestino R, Prazeres H, Lima L, Melo M, da Rocha AG, Preto A (2013). Frequency of TERT promoter mutations in human cancers. Nat Commun.

[13] Huang FW, Hodis E, Xu MJ, Kryukov GV, Chin L, Garraway LA (2013). Highly recurrent TERT promoter mutations in human melanoma. Science.

[14] Hsu CP, Hsu NY, Lee LW, Ko JL (1990). Ets2 binding site single nucleotide polymorphism at the hTERT gene promoter--effect on telomerase expression and telomere length maintenance in non-small cell lung cancer. Eur J Cancer Oxf Engl.

[15] Xu D, Dwyer J, Li H, Duan W, Liu JP (2008). Ets2 maintains hTERT gene expression and breast cancer cell proliferation by interacting with c-Myc. J Biol Chem.

[16] Park CK, Lee SH, Kim JY, Kim JE, Kim TM, Lee ST, Choi SH, Park SH, Kim IH (2014). Expression level of hTERT is regulated by somatic mutation and common single nucleotide polymorphism at promoter region in glioblastoma. Oncotarget.

[17] Nabholtz JM, Abrial C, Mouret-Reynier MA, Dauplat MM, Weber B, Gligorov J, Forest AM, Tredan O, Vanlemmens L, Petit T, Guiu S, Van Praagh I, Jouannaud C (2014). Multicentric neoadjuvant phase II study of panitumumab combined with an anthracycline/taxane-based chemotherapy in operable triple-negative breast cancer: identification of biologically defined signatures predicting treatment impact. Ann Oncol Off J Eur Soc Med Oncol ESMO.

[18] Nabholtz JM, Chalabi N, Radosevic-Robin N, Dauplat MM, Mouret-Reynier MA, Van Praagh I, Servent V, Jacquin JP, Benmammar KE, Kullab S, Bahadoor MRK, Kwiatkowski F, Cayre A (2016). Multicentric neoadjuvant pilot Phase II study of cetuximab combined with docetaxel in operable triple negative breast cancer. Int J Cancer.

[19] Bièche I, Noguès C, Paradis V, Olivi M, Bedossa P, Lidereau R, Vidaud M (2000). Quantitation of hTERT gene expression in sporadic breast tumors with a real-time reverse transcription-polymerase chain reaction assay. Clin Cancer Res Off J Am Assoc Cancer Res.

[20] Brennan CW, Verhaak RGW, McKenna A, Campos B, Noushmehr H, Salama SR, Zheng S, Chakravarty D, Sanborn JZ, Berman SH, Beroukhim R, Bernard B, Wu CJ (2013). The somatic genomic landscape of glioblastoma. Cell.

[21] Labussière M, Di Stefano AL, Gleize V, Boisselier B, Giry M, Mangesius S, Bruno A, Paterra R, Marie Y, Rahimian A, Finocchiaro G, Houlston RS, Hoang-Xuan K (2014). TERT promoter mutations in gliomas, genetic associations and clinico-pathological correlations. Br J Cancer.

[22] Rachakonda PS, Hosen I, de Verdier PJ, Fallah M, Heidenreich B, Ryk C, Wiklund NP, Steineck G, Schadendorf D, Hemminki K, Kumar R (2013). TERT promoter mutations in bladder cancer affect patient survival and disease recurrence through modification by a common polymorphism. Proc Natl Acad Sci U S A.

[23] Ko E, Seo HW, Jung ES, Kim BH, Jung G (2016). The TERT promoter SNP rs2853669 decreases E2F1 transcription factor binding and increases mortality and recurrence risks in liver cancer. Oncotarget.

[24] Hosen I, Rachakonda PS, Heidenreich B, de Verdier PJ, Ryk C, Steineck G, Hemminki K, Kumar R (2015). Mutations in TERT promoter and FGFR3 and telomere length in bladder cancer. Int J Cancer.

[25] Hosen I, Rachakonda PS, Heidenreich B, Sitaram RT, Ljungberg B, Roos G, Hemminki K, Kumar R (2015). TERT promoter mutations in clear cell renal cell carcinoma. Int J Cancer.

[26] Mosrati MA, Malmström A, Lysiak M, Krysztofiak A, Hallbeck M, Milos P, Hallbeck AL, Bratthäll C, Strandéus M, Stenmark-Askmalm M, Söderkvist P (2015). TERT promoter mutations and polymorphisms as prognostic factors in primary glioblastoma. Oncotarget.

[27] Mosrati MA, Willander K, Falk IJ, Hermanson M, Höglund M, Stockelberg D, Wei Y, Lotfi K, Söderkvist P (2015). Association between TERT promoter polymorphisms and acute myeloid leukemia risk and prognosis. Oncotarget.

[28] Zhang A, Zheng C, Lindvall C, Hou M, Ekedahl J, Lewensohn R, Yan Z, Yang X, Henriksson M, Blennow E, Nordenskjöld M, Zetterberg A, Björkholm M (2000). Frequent amplification of the telomerase reverse transcriptase gene in human tumors. Cancer Res.

[29] Xie H, Liu T, Wang N, Björnhagen V, Höög A, Larsson C, Lui WO, Xu D (2014). TERT promoter mutations and gene amplification: promoting TERT expression in Merkel cell carcinoma. Oncotarget.

[30] Zhu CQ, Cutz JC, Liu N, Lau D, Shepherd FA, Squire JA, Tsao MS (2006). Amplification of telomerase (hTERT) gene is a poor prognostic marker in non-small-cell lung cancer. Br J Cancer.

[31] Zhang A, Zheng C, Hou M, Lindvall C, Wallin KL, Angström T, Yang X, Hellström AC, Blennow E, Björkholm M, Zetterberg A, Gruber A, Xu D (2002). Amplification of the telomerase reverse transcriptase (hTERT) gene in cervical carcinomas. Genes Chromosomes Cancer.

[32] Palmqvist R, Zhang A, Xu D, Golovleva I, Norrback KF, Gruber A, Oberg A, Stenling R, Roos G (2005). hTERT gene copy number is not associated with hTERT RNA expression or telomerase activity in colorectal cancer. Int J Cancer.

[33] Diaz A, Puig-Butillé JA, Muñoz C, Costa D, Díez A, Garcia-Herrera A, Carrera C, Badenas C, Solé F, Malvehy J, Puig S, Alos L (2014). TERT gene amplification is associated with poor outcome in acral lentiginous melanoma. J Am Acad Dermatol.

[34] Ruden M, Puri N (2013). Novel anticancer therapeutics targeting telomerase. Cancer Treat Rev.

[35] Latil A, Vidaud D, Valéri A, Fournier G, Vidaud M, Lidereau R, Cussenot O, Biàche I (2000). htert expression correlates with MYC over-expression in human prostate cancer. Int J Cancer.

[36] Wang J, Xie LY, Allan S, Beach D, Hannon GJ (1998). Myc activates telomerase. Genes Dev.

[37] Dwyer J, Li H, Xu D, Liu JP (2007). Transcriptional regulation of telomerase activity: roles of the the Ets transcription factor family. Ann N Y Acad Sci.

[38] Li H, Xu D, Li J, Berndt MC, Liu JP (2006). Transforming Growth Factor β Suppresses Human Telomerase Reverse Transcriptase (hTERT) by Smad3 Interactions with c-Myc and the hTERT Gene. J Biol Chem.

[39] Koh CM, Khattar E, Leow SC, Liu CY, Muller J, Ang WX, Li Y, Franzoso G, Li S, Guccione E, Tergaonkar V (2015). Telomerase regulates MYC-driven oncogenesis independent of its reverse transcriptase activity. J Clin Invest.

[40] Chen Y, Yang Y, van Overbeek M, Donigian JR, Baciu P, de Lange T, Lei M (2008). A shared docking motif in TRF1 and TRF2 used for differential recruitment of telomeric proteins. Science.

[41] Wolfer A, Ramaswamy S (2011). MYC and metastasis. Cancer Res.

[42] Wolfer A, Wittner BS, Irimia D, Flavin RJ, Lupien M, Gunawardane RN, Meyer CA, Lightcap ES, Tamayo P, Mesirov JP, Liu XS, Shioda T, Toner M (2010). MYC regulation of a “poor-prognosis” metastatic cancer cell state. Proc Natl Acad Sci U S A.

[43] Penault-Llorca F, Abrial C, Raoelfils I, Cayre A, Mouret-Reynier MA, Leheurteur M, Durando X, Achard JL, Gimbergues P, Chollet P (2008). Comparison of the prognostic significance of Chevallier and Sataloff's pathologic classifications after neoadjuvant chemotherapy of operable breast cancer. Hum Pathol.

[44] Poncet D, Belleville A, t’kint de Roodenbeke C, Roborel de Climens A, Ben Simon E, Merle-Beral H, Callet-Bauchu E, Salles G, Sabatier L, Delic J, Gilson E (2008). Changes in the expression of telomere maintenance genes suggest global telomere dysfunction in B-chronic lymphocytic leukemia. Blood.

[45] Véronèse L, Tournilhac O, Callanan M, Prie N, Kwiatkowski F, Combes P, Chauvet M, Davi F, Gouas L, Verrelle P, Guièze R, Vago P, Bay JO (2013). Telomeres and chromosomal instability in chronic lymphocytic leukemia. Leukemia.

[46] Mitas M, Mikhitarian K, Walters C, Baron PL, Elliott BM, Brothers TE, Robison JG, Metcalf JS, Palesch YY, Zhang Z, Gillanders WE, Cole DJ (2001). Quantitative real-time RT-PCR detection of breast cancer micrometastasis using a multigene marker panel. Int J Cancer.

[47] Niel F, Martin J, Dastot-Le Moal F, Costes B, Boissier B, Delattre V, Goossens M, Girodon E (2004). Rapid detection of CFTR gene rearrangements impacts on genetic counselling in cystic fibrosis. J Med Genet.

[48] Pebrel-Richard C, Kemeny S, Gouas L, Eymard-Pierre E, Blanc N, Francannet C, Tchirkov A, Goumy C, Vago P (2012). An atypical 0.8 Mb inherited duplication of 22q11.2 associated with psychomotor impairment. Eur J Med Genet.

