# Supplementary material for: TERT promoter status and gene copy number gains: effect on TERT expression and association with prognosis in breast cancer
Source: Oncotarget. 2017 Aug 24;8(44):77540–51. doi: 10.18632/oncotarget.20560 (PMC5652798; doi:10.18632/oncotarget.20560)
Supplement: Supplementary file 1 [file oncotarget-08-77540-s001.pdf]

TERT promoter status and gene copy number gains: effect on *TERT* expression and association with prognosis in breast cancer

SUPPLEMENTARY MATERIALS

Quantification of *TERT* gene copy number by QMF-PCR

QMF-PCR was performed on a TProfessional thermocycler (Biometra, Archamps, France). Primer sequences for *TERT* and control genes are given below.

The forward primers were labeled with fluorescent phosphoramidite 6-FAM dye and all the primers were HPLC-purified. The PCR reactions were performed with the QIAGEN Multiplex PCR kit (Qiagen, Courtaboeuf, France), with 40 ng of genomic DNA and a mix of primers. The reaction started with an initial denaturation of 15 min at 95°C, followed by 23 cycles at 95°C for 30s, 60°C for 90s and 72°C for 90s, and a final extension of 10 min at 72°C. One µl of the PCR products was then added to 10 µl of formamide and 0.35 µl of GeneScan-600 LIZ size standard (Applied Biosystems, Courtaboeuf, France). The fluorescent PCR products were heat-denatured, chilled on ice, and separated on an 8-capillary sequencer (3500 Genetic Analyzer, Applied Biosystems). The results were processed with GeneMapper 5 software (Applied Biosystems) to obtain electrophoregrams for each sample. Each product was identified by its size, and the peak area values were imported into an Excel (Microsoft) spreadsheet and the copy number of each fragment was determined by calculating a dosage quotient (DQ) for each fragment.

Gene		Sequence 5'-3'	Product size
**TERT-3'UTR**	F	AGCTTTTCCTCACCAGGAGC	100 bp
	R	AGGGGTGAACAATGGCGAAT	
**TERT-Exon 9**	F	TTTGCCGGCTGAATGGTAGA	110 bp
	R	GTGCTGGACATGCGTACACT	
**PVRL1**	F	TGACCACTTGCTGCCATCCTCCT	141 bp
	R	AGCGGGTTGGCAAAGCTGCA	
**BOD1L**	F	GCAACTCACACATGGAGTCCGCA	161 bp
	R	GCCTCCGCTTTCAGGCAAAGT	
**RET**	F	ATTGGCCTGTCTGCTCTTCC	168 bp
	R	GAATTGGACCCAGGCACTCA	
**ZNF638**	F	TGAAGTACATTGAAACAACACCTCT	191 bp
	R	ATGCCTATTTCTATATGTGTGCCA	
**AGBL2**	F	ACCCTCAATGCCTGTTGAATGGC	203 bp
	R	GCTCCCTGGCCTCTCCCATG	
**CFTR**	F	TCCTATGACCCGGATAACAAGGAGG	226 bp
	R	ATATATGTGCCATGGGGCCTGTGC	
**POR**	F	TCAGCCTCCTCTCTCTGTCC	248 bp
	R	TTTCGCAGTGCTTCCTGTGA	
